# Hypothalamus volumes and mental health in children and adolescents

**DOI:** 10.3389/fnins.2026.1757229

**Published:** 2026-03-10

**Authors:** Madeson Todd, Bryce Geeraert, Kirk Graff, Catherine Lebel, Kathryn Y. Manning

**Affiliations:** 1 Department of Radiology, Cumming School of Medicine, University of Calgary, Calgary, AB, Canada; 2 Department of Family Practice, University of British Columbia, Vancouver, BC, Canada; 3 Owerko Centre, University of Calgary, Calgary, AB, Canada; 4 Alberta Children’s Hospital Research Institute, Calgary, AB, Canada

**Keywords:** adolescents, brain, children, hypothalamus, mental health, MRI, stress-related mental disorders

## Abstract

**Introduction:**

The hypothalamus-pituitary–adrenal (HPA) axis plays an important role in regulating behavior, neuroplastic responses to the environment during childhood and adolescent development, and highly implicated in stress-related mental disorders. However, due to the small size of hypothalamic structures and the limited availability of automated segmentation tools, there are relatively few neuroimaging studies examining hypothalamic involvement in mental health in human populations. Using a semi-automated segmentation approach, we conducted an exploratory study examining associations between hypothalamic volume and mental health-related behaviors in typically developing youth.

**Methods:**

T1-weighted magnetic resonance imaging (MRI) scans and behavioral measures [Behavioral Assessment System for Children-2 Parent Report Scale (BASC-2 PRS)] were collected from 71 youth (aged 6–16 years). T1-weighted MRI data were quality checked and processed, and hypothalamic volumes semi-automatically delineated. Left, right and total hypothalamus volumes were tested across age using linear mixed effects models, as well as tested separately for associations with clinical T scores using a general linear model.

**Results:**

Left (*T* = −2.0; *p* = 0.04) and total (*T* = −2.6, *p* = 0.01) hypothalamic volume decreased with age. A trend-level association was observed between left hypothalamic volume and adaptability scores (*T* = 1.8; *p* = 0.08), which did not reach conventional statistical significance. No significant associations were observed for internalizing or externalizing scores.

**Conclusion:**

Decreased ability to adapt to one’s environment may be a predictor of mental illness. In this exploratory study, we observed significantly decreasing hypothalamus volume across this age range. There was a trend-level association between hypothalamic volume and adaptability, suggesting that structural variation in this region may be relevant to stress-related functioning in youth. However, this finding should be interpreted cautiously and requires replication in larger, longitudinal samples.

## 1 Introduction

The transition from childhood to adolescence is a highly dynamic period of neural organization and maturation (Shaw et al., 2020) including neural pruning, myelination, and cytoskeletal alterations (Lebel and Deoni, 2018; Moura et al., 2016; Shaw et al., 2020). Disruption of these processes can predispose the individual to behavioral difficulties and make them more vulnerable to developing mental health disorders (Baker et al., 2011; Paus et al., 2008).

Half of all mental illnesses begin by age 15 years; however, most go undetected and/or untreated for several years (McGrath et al., 2023; World Health Organization, 2019). Identifying markers of future mental disorders would be helpful for earlier diagnosis and may shed light on preventative treatment options. Studying children who exhibit subclinical behaviors may be useful for identifying early indicators of mental health problems that may not otherwise become clinically apparent until later (Baker et al., 2011).

The hypothalamic–pituitary–adrenal (HPA) axis plays a central role in stress physiology and behavioral regulation, and its developmental calibration during childhood and adolescence influences vulnerability to later mental health outcomes. Recent work indicates that developmental changes in HPA axis activity are linked to individual differences in stress responsivity and associations with psychopathology in youth (Filetti et al., 2024; Leroux et al., 2023), and that exposure to childhood adversity can alter HPA axis functioning in age-dependent ways (Niu et al., 2025).

The HPA axis is particularly relevant to mental health, as neurons in the paraventricular nucleus of the hypothalamus secrete corticotropic releasing hormone (CRH), which stimulates the anterior pituitary to release adrenocorticotropic hormone and ultimately causes the liberation of cortisol secretion (Meynen et al., 2007). Altered negative feedback with this system has been observed in stress related mental disorders such as depression and generalized anxiety disorder (Faravelli et al., 2012; Varghese and Brown, 2001), including reduced sensitivity to glucocorticoid suppression following dexamethasone administration (Schweizer et al., 1986; Strawbridge et al., 2017). Chronic hyperactivity of CRH neurons and prolonged exposure to glucocorticoids may contribute to structural alterations within the hypothalamus through neurotoxicity, atrophy of dendritic processes, and/or neuronal death (Bachis et al., 2008; Colla et al., 2007).

While other brain markers of depression and anxiety have been studied—particularly the prefrontal cortex, amygdala, and striatum (Baker et al., 2015; Blair and Zhang, 2020; Fonseka et al., 2018; Hammoud et al., 2024; Johanson et al., 2020; Noordermeer et al., 2016; Teeuw et al., 2022)—little research has looked at relationships with the structural components of the HPA axis, despite evidence of abnormal function in stress-related mental disorders (Fischer et al., 2017; Ha et al., 2019; McCormick and Mathews, 2010; Sher, 2007; Varghese and Brown, 2001). Overall, studies are in good agreement that there is hyperactivity in the HPA axis in individuals with stress related mental disorders, and have shown that there are higher rates of relapse of such disorders in individuals where that hyperactivity persists (Chang et al., 2022; Holsen et al., 2013; Juruena et al., 2020; Menke et al., 2018). A structural biomarker indicating predisposition to mental illness could inform early diagnosis and intervention strategies—an important objective as structural measures such as regional brain volume are often more stable and less state-dependent than functional activity (Osmanlıoğlu et al., 2020).

A handful of volumetric MRI studies have analyzed the pituitary and adrenal glands. Study results vary, with some reporting enlarged pituitaries and hyperactive adrenal glands in depression and anxiety (Kessing et al., 2011; Lorenzetti et al., 2009; MacMaster et al., 2006; Macmaster et al., 2008; MacMaster and Kusumakar, 2004; Rama Krishnan et al., 1991; Schwartz et al., 1997), while others found smaller pituitaries, specifically in individuals with bipolar disorder (Delvecchio et al., 2018). Despite the hypothalamus being a key component of the HPA axis -and its abnormal function in stress-related mental disorders validated through histological, post-mortem, and animal—it is often omitted in human neuroimaging research due to its small size and challenging anatomy (Austin et al., 2003; Faravelli et al., 2012; Fischer et al., 2017; Ha et al., 2019, p. 29; McCormick et al., 2010; Purba et al., 1996; Raadsheer et al., 1994, 1995; Sher, 2007; Varghese and Brown, 2001).

Only a few studies have examined hypothalamic structure in relation to mental health. One showed smaller hypothalamic volumes in adults with generalized anxiety disorder (GAD) (Terlevic et al., 2013). More recently, a longitudinal study reported that smaller right hypothalamic volumes measured 2 weeks after adult trauma mediated the relationship between adverse childhood events (ACEs) and PTSD symptom severity 1 year later, suggesting that early adversity may leave lasting structural changes in the hypothalamus that increase vulnerability to future stress-related disorders (Xie et al., 2023). These findings highlight the hypothalamus as a potentially important region in the development of anxiety and PTSD symptoms. However, further research is needed to explore its role across a broader spectrum of mental health outcomes, including subclinical symptoms that may precede diagnosable disorders.

Evaluation of hypothalamic development could be useful in identifying children at risk of developing mental disorders, which could inform preventative treatments. Thus, the purpose of this exploratory study was to (a) characterize the volume of the hypothalamus during typical development and (b) explore whether mental health-related behaviors related to the volume of the hypothalamus. We predicted smaller hypothalamus volume in individuals with more severe mental health related behaviors.

## 2 Materials and methods

### 2.1 Study design and participants

Seventy-one children and adolescents (6–16 years, mean age = 10.73, SD = 2.56, male = 36, female = 35) participated in magnetic resonance imaging (MRI) at the Alberta Children’s Hospital in Calgary, Alberta, Canada. Inclusion criteria were: (1) uncomplicated birth between 37 and 42 weeks’ gestation, (2) no history of developmental disorder, psychiatric disease, or reading difficulty, (3) no history of neurosurgery, and (4) no contraindications to MRI. Parent-reported behavioral functioning was assessed using the Behavioral Assessment System for Children, Second Edition Parent Rating Scale (BASC-2-PRS) (Xie et al., 2023) Participants provided informed assent, and their guardians written informed consent. The study was approved by the local research ethics board (CHREB, ID: REB13-1346).

Participants were classified into low-risk and high-risk groups based on T-scores for Externalizing behavior, Internalizing behavior, and Adaptability. For Externalizing and Internalizing scales, higher scores represent more problematic behaviors, with scores of ≥60 defined as high risk and scores ≥70 clinically relevant (Ensign et al., 2012). In contrast, Adaptability scores are at risk when <40, and not at risk when ≥40 (Reynolds and Kamphaus, 2015).

### 2.2 Image acquisition and preprocessing

MRI scans were collected using a 32-channel head coil on a GE 3 T Discovery MR750w (GE, Milwaukee, WI). T1-weighted images were acquired using a fast-spoiled gradient echo (FSPGR) sequence, 210 axial slices; 0.9 × 0.9 × 0.9 mm resolution, repetition time (TR) = 8.23 ms, echo time (TE) = 3.76 ms, flip angle = 12°, matrix size = 512 × 512, inversion time = 540 ms. T1-weighted data were quality-checked and initially corrected for intensity inhomogeneity using N4BiasFieldCorrection in Advanced Normalization Tools (ANTs).

### 2.3 Segmenting and volumizing the hypothalamus

**Figure 1:**
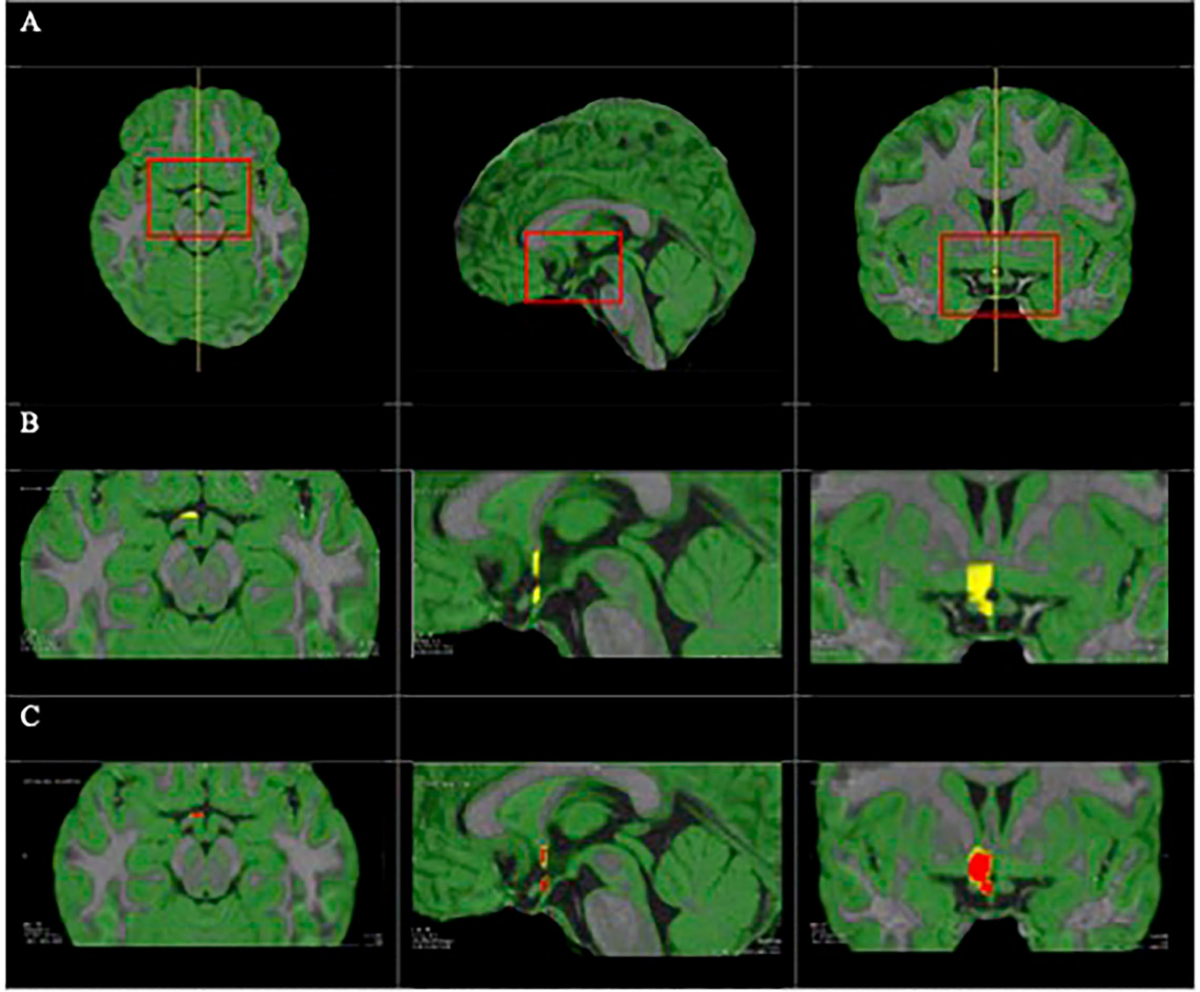
**(A)** Triplanar view with T1-weighted image and GM reference as a green overlay; full view (left) and zoomed in on the hypothalamus (right). Volumetry of preoptic hypothalamus: ROI in yellow **(B)** and seed growing step in red **(C)**.

T1-weighted images then underwent further preprocessing in FSL, which included spatial smoothing, motion correction, distortion correction, and resampling to 1 × 1 × 1 mm voxels. The hypothalamus in each hemisphere was manually delineated in MeVisLab software following an algorithm developed by Wolff et al. (2018). Briefly, the hypothalamus was divided into four regions of interest (ROIs) per hemisphere: preoptic, intermediate superior, intermediate inferior and posterior. Landmarks were manually placed on voxels that outline each ROI (Figure 1). A voxel within that region was then selected, and the software automatically fills in the ROI indicated by the manually placed boundaries. Voxels at the boundary are excluded according to the probability thresholds, which were set at 0.90, 0.85. 0.85, and 0.80 for the preoptic, intermediate superior, intermediate inferior and posterior hypothalamic areas, respectively, to achieve the best segmentation. Following delineation, hypothalamus volume was calculated in mm^3^ for the left, right, and total hypothalamus for each participant. Total intracranial volumes were calculated using FreeSurfer version 7.1.0.

### 2.4 Data characteristics and statistical analysis

Linear mixed effects models were analyzed using Matlab version R2021b. We assessed the relationship between age and total, left, and right hypothalamic volumes separately using a general linear model, controlling for sex and intracranial volume.

Subsequently, left, right, and total hypothalamic volumes were tested separately using a general linear model to predict three BASC-2-PR Internalizing, Externalizing, and Adaptive T-scores, while controlling for age, sex, and total intracranial volume for a total of 9 models. SPSS Statistics version 29.0.0.0 was used to perform unpaired *t*-tests for hypothalamus volume differences between low-risk and high-risk group (<60 and ≥60 for Internalizing/Externalizing T scores and are >40 and ≤40 for Adaptability) (Reynolds and Kamphaus, 2015). Due to minimal high risk Adaptability T Scores, (only 7 of the 71 Adaptability T scores < 40), the scores were compared using Welsch’s *t*-tests.

## 3 Results

### 3.1 Sample characteristics

Seventy-one children and adolescents aged 6–16 years (mean age = 10.73 years, SD = 2.56; 36 males, 35 females) participated in magnetic resonance imaging at the Alberta Children’s Hospital in Calgary, Alberta, Canada. Parent-reported behavioral functioning was assessed using the Behavioral Assessment System for Children, Second Edition Parent Rating Scale (BASC-2-PRS). Descriptive statistics for demographic characteristics and hypothalamic volumes are presented in Table 1. Behavioral scores spanned a range of symptom severity, allowing examination of associations between hypothalamic volume and behavioral risk across the sample.

**Table 1:** Demographic characteristics and hypothalamic volumes of the study sample (*N* = 71).

Variable	Mean	SD	Minimum	Maximum
Age (years)	10.73	2.56	6.05	16.06
Male/Female (*n*)	36/35	–	–	–
Total hypothalamic volume (mm^3^)	1668.09	63.19	1,546	1837
Right hypothalamic volume (mm^3^)	843.65	47.93	742	949
Left hypothalamic volume (mm^3^)	838.76	37.16	722	914

Values are presented as mean, standard deviation (SD), minimum, and maximum, where applicable. Hypothalamic volumes are reported in cubic millimeters (mm^3^).

### 3.2 Hypothalamus volumes and age

**Figure 2:**
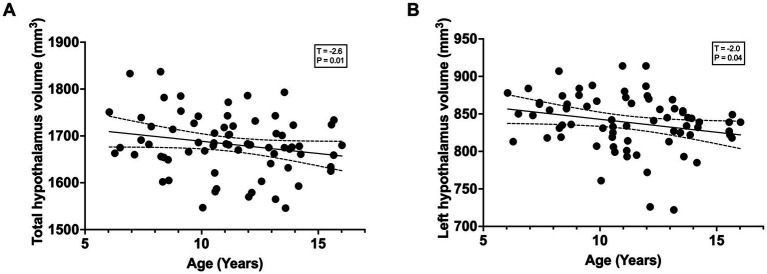
**(A)** Total hypothalamic volume showed a significant negative association with age (*T*–2.6, *P*-0.01). **(B)** Left hypothalamic volume also demonstrated a significant negative association with age (*T*–2.0, *P*-0.04). Lines represent linear regression fits with 95% confidence intervals.

The average hypothalamus volume was 1,668 mm^3^ (SD = 69.30 mm^3^, min = 1,456 mm^3^, max = 1,837 mm^3^). There was a significant relationship between left hypothalamic volume and age (Figure 2A) (*T* = −2.0; *p* = 0.04) and total hypothalamic volume and age (Figure 2B) (*T* = −2.6, *p* = 0.01), controlling for sex and total intracranial volume. The same relationship was not observed between the right hypothalamic volume (*T* = −1.8; *p* = 0.07) and age.

### 3.3 BASC-2 scores and relations to hypothalamus volume

**Figure 3:**
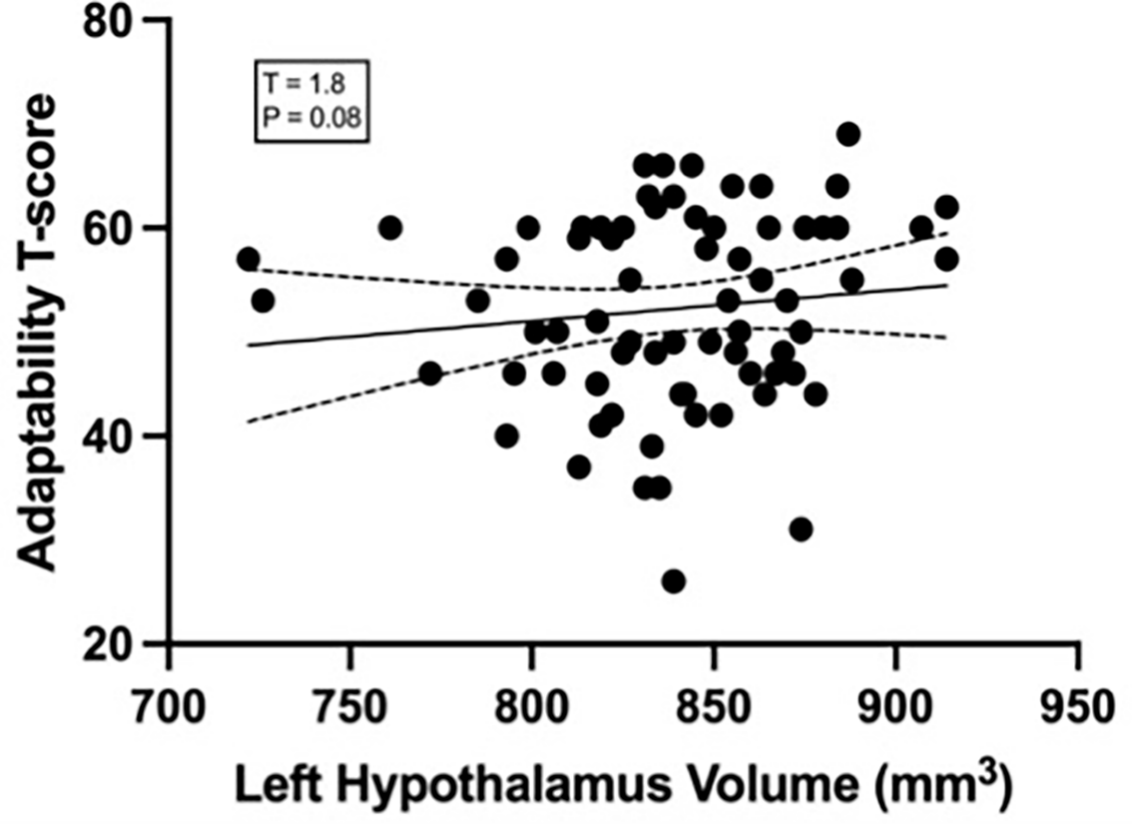
The relationship between the left hypothalamus volume (mm) and adaptability. *T*-score residuals were small (*T* = 1.8, *p* = 0.08, *r* = 0.05).

No significant associations were observed between hypothalamic volumes and BASC-2 Internalizing, Externalizing, or Adaptability scores (Supplementary Table 1), although a trend-level association was observed between Adaptability scores and left hypothalamic volume (Figure 3), which was not statistically significant (*T* = −1.8, *p* = 0.08).

Group comparisons based on established clinical cut-offs for Externalizing, Internalizing, and Adaptability T-scores are presented in Table 2. No significant differences in total, left, or right hypothalamic volumes were observed between low- and high-risk groups (all *p* > 0.05). Full results and visualizations are presented in Supplementary Figure 1.

**Table 2:** Comparison of hypothalamic volumes and BASC-2 T-scores between low- and high-risk groups for (A) Externalizing behavior, (B) Internalizing behavior, and (C) Adaptability.

A. Externalizing behaviors			
Measure	Low-risk (*T* < 60, *n* = 57)	High-risk (*T* ≥ 60, *n* = 14)	*p*-value
Externalizing T-score	48.58 ± 5.62	66.57 ± 6.41	< 0.001
Total volume (mm^3^)	1684.25 ± 65.43	1674.93 ± 54.62	0.59
Right volume (mm^3^)	846.51 ± 49.13	832.00 ± 42.28	0.28
Left volume (mm^3^)	837.74 ± 39.69	842.93 ± 25.02	0.55

B. Internalizing behaviors			
Measure	Low-risk (*T* < 60, *n* = 56)	High-risk (*T* ≥ 60, *n* = 15)	*p*-value
Internalizing T-score	47.48 ± 6.92	68.80 ± 5.89	< 0.001
Total volume (mm^3^)	1683.21 ± 65.44	1679.40 ± 55.96	0.82
Right volume (mm^3^)	844.91 ± 47.35	838.93 ± 51.47	0.69
Left volume (mm^3^)	838.30 ± 40.00	840.47 ± 24.79	0.80

C. Adaptability			
Measure	Low-risk (*T* > 40, *n* = 64)	High-risk (*T* ≤ 0, *n* = 7)	*p*-value
Adaptability T-score	54.14 ± 7.51	34.71 ± 4.86	< 0.001
Total volume (mm^3^)	1684.91 ± 65.83	1659.57 ± 20.26	0.033
Right volume (mm^3^)	845.31 ± 49.24	828.43 ± 32.27	0.247
Left volume (mm^3^)	839.59 ± 38.32	831.14 ± 24.84	0.442

Low- and high-risk groups were defined as follows: for Externalizing and Internalizing scales, low risk = *T* < 60 and high risk = *T* ≥ 60; for Adaptability, high risk = *T* ≤ 40 and low risk = *T* > 40. Values are presented as mean ± SD. Group comparisons were conducted using Welch’s *t*-tests.

## 4 Discussion

In this study, we found age-related decreases in left hypothalamic volume. The observed mean hypothalamic volume is consistent with previous histological studies and neuroimaging studies reporting similar volumetric ranges in pediatric and adult samples (Goldstein et al., 2007; Schindler et al., 2013; Wolff et al., 2018) Although not significant, there was a trend toward larger left hypothalamic volumes in youth with increased adaptability scores (i.e., more adaptable), suggesting hypothalamic volumes may support better emotional regulation and adaptability to stress. This trend-level association should be interpreted cautiously, as it did not survive correction for multiple comparisons and may reflect exploratory signal rather than a robust effect. To the best of our knowledge, this is the first study to evaluate how structural differences of the hypothalamus correlate with behavioral measures in a preclinical youth sample across a broad spectrum of stress-related mental health outcomes.

Although hypothalamus volume decreased with age—consistent with normative synaptic pruning and neurodevelopment (Konrad et al., 2013; Tierney and Nelson, 2009)—our trend-level finding that smaller volumes were also associated with lower adaptability scores presents an interesting tension. While coping and adaptability generally improve with age, this apparent contradiction may reflect region-specific developmental dynamics. The hypothalamus undergoes early maturation and may be especially sensitive to early life stress (Maniam et al., 2014). Thus, individual differences in hypothalamus volume—particularly when smaller than expected for one’s age—may reflect stress-related neurodevelopmental vulnerabilities rather than typical developmental patterns. It is also possible that while the hypothalamus plays a prominent role in stress regulation in childhood, other brain regions such as the prefrontal cortex or anterior cingulate may assume greater responsibility for adaptive coping as development progresses (Griffin, 2017; Kolk and Rakic, 2022). Future research examining age-normed trajectories of hypothalamus volume in relation to behavior may help disentangle these developmental effects.

With that in mind, our findings of decreased left hypothalamic volume in youth with decreased risk adaptability T scores—reflecting poorer perceived coping ability—adds to the evidence of stress-related HPA axis alterations, which hypothesize hypothalamic atrophy following prolonged HPA dysregulation and glucocorticoid exposure. This aligns with one previous study that found smaller hypothalamic size in children with autism compared to controls (Kurth et al., 2011), who also tend to have poorer adaptability, and with literature linking lower adaptability to increased vulnerability for developing mood disorders such as anxiety and depression (Baker et al., 2011). In this context, reduced hypothalamic volume may reflect heightened stress sensitivity and may serve as a potential early biomarker of stress-related mental health disorders. Exploring such measurable biomarkers is valuable in understanding the etiology and early detection of depression and anxiety in youth.

While the association between adaptability and hypothalamic volume was specific to the left hemisphere, trends in the right hypothalamus followed a similar direction, albeit less robustly. This may reflect true asymmetry in structural change, or variation not fully captured in our sample. Brain lateralization is well-established for functions such as language (Duboc et al., 2015), and emerging evidence suggests that the hypothalamus may also exhibit hemisphere-specific roles, particularly in reproductive function and energy regulation (Kiss et al., 2020). These processes are modulated through circuits involving hormonal regulation, autonomic activity, and homeostatic drives, which may show hemisphere-specific organization and asymmetry (Duboc et al., 2015). Although mechanisms remain unclear, the observed left-sided decrease aligns with known patterns of synaptic pruning and intracortical myelination during development (Gogtay and Thompson, 2010; Paus, 2005). As these processes occur bilaterally (Giorgio et al., 2010), the lack of a significant correlation in the right hypothalamus may reflect both a weaker or delayed developmental change in that hemisphere.

Isıklar et al. (2022) reported that hypothalamic volume increases logarithmically during early childhood, with rapid growth during the first 2 years. They also found larger hypothalamic volumes in males, and consistent asymmetry favoring the right side. Our study corroborates this asymmetry but observed a negative correlation between left and total hypothalamic volume and age, potentially reflecting different biological changes in the age range studied here. This may reflect methodological differences, sample characteristics, or the unique stress-behavior focus of our cohort. Given that youth with poor adaptability may already be on a trajectory toward mental health challenges, our findings may reflect early structural differences associated with chronic stress exposure or vulnerability.

Although we did not find significant associations between hypothalamic volume and internalizing or externalizing symptoms, previous studies have shown that externalizing behaviors—such as hyperactivity, aggression, and delinquency—are linked to structural and functional differences in brain regions including the prefrontal cortex, amygdala, and striatum (Baker et al., 2015; Blair and Zhang, 2020; Johanson et al., 2020; Noordermeer et al., 2016; Teeuw et al., 2022). Specifically, studies show a reduction in gray matter with higher-risk externalizing behaviors that goes beyond synaptic pruning in a typically developing brain (Baker et al., 2015; Bayard et al., 2020; Durham et al., 2021; Raine et al., 2000; Teeuw et al., 2022; Waller et al., 2020). Likewise, internalizing behaviors, such as depression and anxiety, have also been linked to a reduction in gray matter (Ancelin et al., 2019; Kandilarova et al., 2019; Kraus et al., 2017; Zhao et al., 2023). Only one study has reported smaller hypothalamic volumes in adults with generalized anxiety disorder (Terlevic et al., 2013), but it focused exclusively on adults and did not assess a broader spectrum of stress-related mental health outcomes, highlighting the need for further research into this structure’s role across development. Our null findings may reflect regional specificity, where the hypothalamus is less sensitive to subclinical behavioral variation during this developmental stage. It is also possible that associations with hypothalamic volume become more pronounced later in development or in populations with broader ranges of behavioral symptoms. Further research is warranted to explore hypothalamic volume in relation to both externalizing and internalizing behaviors, across a broader range of clinical and developmental contexts.

The hypothalamus plays a central role in regulating the HPA axis, and chronic activity—such as hyperactivity of CRH neurons—may lead to desensitization and atrophy due to prolonged glucocorticoid exposure. Hyperactivity of the HPA axis is a key characteristic and predictor of one’s susceptibility to develop stress-related mental disorders (Colla et al., 2007; Gold et al., 2015; Strawbridge et al., 2017). Given that decreased gray matter volumes are seen in various child psychiatric conditions (ADHD, depression, GAD, bipolar disorder, Ancelin et al., 2019; Delvecchio et al., 2018; Kandilarova et al., 2019; Li et al., 2022; Macmaster et al., 2008; Sassi et al., 2001; Terlevic et al., 2013), our findings contribute to the growing recognition of the hypothalamus as a meaningful structure in the early neurobiology of mental health.

This study also underscores the potential of emerging semi-automated segmentation tools for measuring small brain structures like the hypothalamus, opening new possibilities for early detection. Manual segmentation remains the gold standard, but it is labor-intensive and lacks scalability. At the time of our study, only one group had used automated methods in healthy youth (Isıklar et al., 2022), and none have related these measures to behavioral profiles. Since then, other automated approaches have emerged and show promise for improving segmentation accuracy (Billot et al., 2020; Estrada et al., 2023). Leveraging these tools could facilitate larger, longitudinal studies to map hypothalamic development over time and its relationship to the emergence and progression of stress-related mental health conditions. Incorporating additional modalities, such as diffusion tensor imaging and functional connectivity, may also clarify underlying mechanisms and pathways.

Overall, our findings support the hypothalamus as a potentially important marker of early stress sensitivity, particularly in youth with low adaptability. Future work using improved segmentation methods, diverse populations, and longitudinal designs is critical to clarifying the hypothalamus’ role in mental health trajectories. Identifying early structural biomarkers may ultimately support early detection and personalized interventional during critical periods of brain development. We hope that the findings of this study motivate further investigation of this topic.

### 4.1 Limitations

The modest sample size and narrow range of T-scores may have limited our ability to detect small effects, potentially contributing to the lack of associations. In addition, formal correction for multiple comparisons was not applied in this exploratory study, increasing the possibility of false-positive findings. Participants were generally healthy and within typical developmental ranges, so findings may not generalize to clinical populations with diagnosed psychiatric conditions. Although the use of semi-automated segmentation is a methodological strength, it still depends on high-quality scans and manual input and may not fully capture functional or subregional differences within the hypothalamus. The cross-sectional design also limits conclusions about causality or developmental change. Lastly, we did not include biological stress markers such as cortisol, which could further clarify the relationship between hypothalamic structure and stress sensitivity.

## 5 Conclusion

We found that left and total hypothalamic volume significantly decreased with age. Further, participants with higher-risk scores had insignificant hypothalamic differences, with a trend-level association between adaptability behavior and left hypothalamic volumes. This exploratory finding underscores the importance of studying brain morphology alongside functional architecture. Further investigations of such delicate and complicated structures could provide valuable insight to the etiology of stress-related mental disorders, in turn providing predictive value that may benefit diagnostic fields. Overall, our findings and others suggest there are complex relationships among across development between brain structure and behavior. Future studies should investigate these brain structures using longitudinal data to fully appreciate the ongoing brain development and to understand the timing of potential early brain differences and their role in behavioral development and mental health.

## Data Availability

Publicly available datasets were analyzed in this study. This data can be found at: https://www.sciencedirect.com/science/article/pii/S2352340920301189.
