# Supplementary figures and images for: Hypothalamus volumes and mental health in children and adolescents
Source: Front Neurosci. 2026 Mar 10;20:1757229. doi: 10.3389/fnins.2026.1757229 (PMC13008893; doi:10.3389/fnins.2026.1757229)

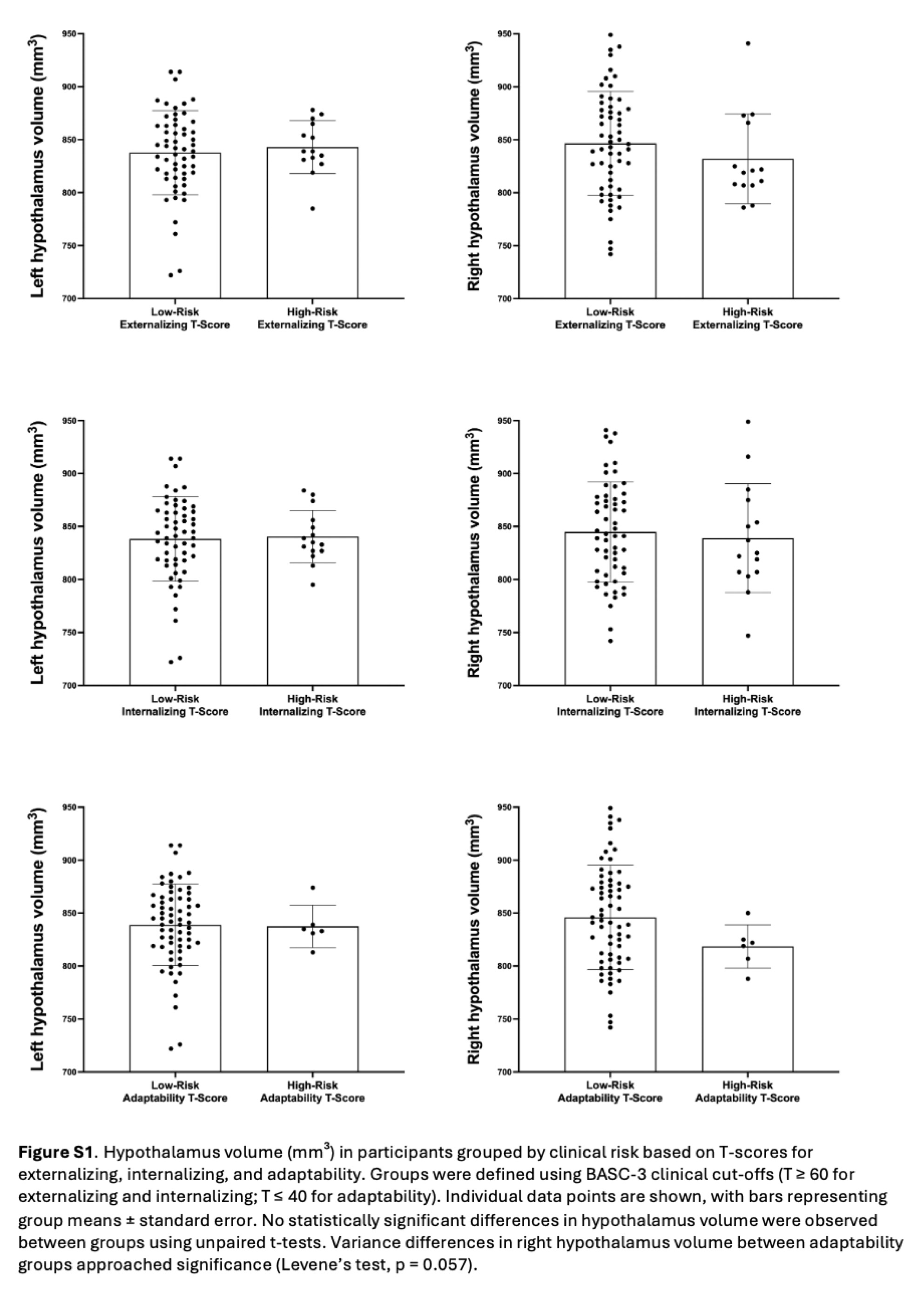

Supplement: Supplementary file 1 [file Image_1.jpeg]
