# Supplementary material for: Hypothalamus volumes and mental health in children and adolescents
Source: Front Neurosci. 2026 Mar 10;20:1757229. doi: 10.3389/fnins.2026.1757229 (PMC13008893; doi:10.3389/fnins.2026.1757229)
Supplement: Supplementary file 2 [file Table_1.pdf]

| Behavioural Measure | Hypothalamus | T | P | $r^2$ |
|---|---|---|---|---|
| BASC-2 Adaptability Scores | Left | 1.8 | 0.08 | 0.05 |
| | Right | 0.6 | 0.5 | 0.04 |
| | Total | 1.2 | 0.2 | 0.05 |
| BASC-2 Internalizing Scores | Left | -0.04 | 0.7 | 0.05 |
| | Right | 0.2 | 0.9 | 0.06 |
| | Total | -0.04 | 0.9 | 0.006 |
| BASC-2 Externalizing Scores | Left | -0.3 | 0.8 | 0.004 |
| | Right | 1.3 | 0.2 | 0.007 |
| | Total | 0.8 | 0.4 | 0.001 |

**Table 2.** BASC-2 scores and relations to hypothalamus volumes. No significant relationships were found between hypothalamic volumes and BASC-2 Adaptability, Internalizing and Externalizing clinical T scores.
